# Holistic processing of fingerprints by expert forensic examiners

**DOI:** 10.1186/s41235-017-0051-x

**Published:** 2017-02-20

**Authors:** Macgregor D. Vogelsang, Thomas J. Palmeri, Thomas A. Busey

**Affiliations:** 1 Indiana University, Bloomington, IN USA; 2 Vanderbilt University, Nashville, TN USA

**Keywords:** Fingerprints, Expertise, Holistic processing, Composite task

## Abstract

Holistic processing is often characterized as a process by which objects are perceived as a whole rather than a compilation of individual features. This mechanism may play an important role in the development of perceptual expertise because it allows for rapid integration across image regions. The present work explores whether holistic processing is present in latent fingerprint examiners, who compare fingerprints collected from crime scenes against a set of standards taken from a suspect. We adapted a composite task widely used in the face recognition and perceptual expertise literatures, in which participants were asked to match only a particular half of a fingerprint with a previous image while ignoring the other half. We tested both experts and novices, using both upright and inverted fingerprints. For upright fingerprints, we found weak evidence for holistic processing, but with no differences between experts and novices with respect to holistic processing. For inverted fingerprints, we found stronger evidence of holistic processing, with weak evidence for differences between experts and novices. These relatively weak holistic processing effects contrast with robust evidence for holistic processing with faces and with objects in other domains of perceptual expertise. The data constrain models of holistic processing by demonstrating that latent fingerprint experts and novices may not substantively differ in terms of the amount of holistic processing and that inverted stimuli actually produced more evidence for holistic processing than upright stimuli. Important differences between the present fingerprint stimuli and those in the literature include the lack of verbal labels for experts and the absence of strong vertical asymmetries, both of which might contribute to stronger holistic processing signatures in other stimulus domains.

## Significance

Fingerprints are currently matched by expert forensic examiners and computers are typically only used to develop candidates for comparison. Accuracy depends on a number of factors, including the quality of the latent print and similarity of the candidate exemplar as suggested by a database search. Efficiency is also important, because many state and local crime labs have long backlogs. These criteria place great weight on the perceptual skills of examiners, who typically undergo extensive multi-year training protocols before they are allowed to report conclusions for their department. Characterizations of the nature of the perceptual and decision-making capacities have the potential to reduce errors, improve training, and increase throughput. The current research addresses the perceptual mechanisms that support expert perceptual decision-making in fingerprint comparisons. We apply a widely-used paradigm, the composite task, because of its rich links to other domains such as musical notation, faces, cars, and novel training stimuli such as greebles. The results test the proposition that experts utilize holistic processing when performing rapid visual analyses of fingerprint-like stimuli. These findings extend the composite task to a new set of perceptual stimuli, which helps clarify the boundaries between stimuli that produce evidence of holistic processing and those that do not.

## Background

Fingerprints as evidence are an effective means to associate an individual to a crime scene, in part because fingerprint impressions are readily available from many surfaces. Fingerprints are unique, even those from identical twins (Srihari, Srinivasan, & Fang, 2008). However, each impression is also unique, due to distortion, visual noise, color inversion, deposition medium, and pressure. Because of this variation in appearance even between impressions from the same finger, all testimony in court is given by human experts. An expert must evaluate the details in agreement between the questioned and exemplar impressions and consider the specificity of these details in order to render one of three decisions: identification (the two impressions are from the same finger), exclusion (the two impressions are from different fingers), or in some labs, inconclusive (in which no determination could be made). Computer databases can be used to develop suspects in a case, but database searches can return large numbers of similar-looking impressions. Thus, the concept of a “match” in forensic identifications actually implies a form of similarity judgment, where the amount of detail must be in sufficient agreement and provide sufficient specificity for the examiner to conclude identification. This is further complicated by the fact that an examiner rarely has an opportunity to examine the impressions of all other individuals who may have had contact with a surface, which requires them to focus on features that might be most rare (Busey, Nikolov, Yu, Emerick, & Vanderkolk, 2016).

These considerations place a burden on the perceptual and visual working memory capacities of human experts. Thompson and Tangen (2014) demonstrated that the nature of expertise among fingerprint examiners might lie in an improved ability to discriminate subtle differences among highly similar non-mated impressions. In their experiments, experts performed much better than novices on a matching task with similar and dissimilar distractors. This held even when a 5-s delay was inserted between two sequentially presented images and inversion surprisingly produced no differences in performance. However, differences between experts and novices on a long-term memory task were mainly due to changes in response criteria, because experts and novices showed equivalent ability to discriminate prints in a long-term memory task. These findings argue for a perceptual basis for the expertise, which may involve extensive perceptual learning in order for an expert to perform competently.

The goal of the present study is to address whether holistic processing might be one mechanism underlying the development of expertise in fingerprint examiners. Holistic processing is a theoretical construct originally used to characterize important differences between face and common object recognition, but has since been used more generally to characterize differences between expert and novice object recognition. While there have been various operational definitions of holistic processing empirically and these have been tied to various mechanistic assumptions theoretically (e.g. Richler, Palmeri, & Gauthier (2012)) they all share the intuitive notion of processing an object as a whole rather than in terms of its constituent parts. For expertise by fingerprint examiners, holistic processing might support the interpretation of individual features in context with their surroundings or allow for localization of target features when doing visual search during a comparison. Below, we briefly describe the process by which fingerprint examiners compare impressions, which serves to illustrate the kind of perceptual decision-making skills that are required. We then discuss the prior literature on the composite task (Gauthier & Bukach, 2007; Hole, 1994; Richler et al., 2012; Young, Hellawell, & Hay, 1987), which has previously been used as one important measure in holistic processing in face recognition and various domains of expertise and which we adapted for our participants.

In the most common framework used by examiners to document the examination process, termed ACE-V (Vanderkolk, 2009), evidence for agreement or disagreement is collected through visual comparison of two impressions and, if no explainable differences are found, and the detail in agreement is considered sufficient, the examiner will conclude “identification.” This reliance on human expertise in comparisons has been highlighted by recent court cases and high-profile errors, which called into question the validity of fingerprint evidence and the ability of experts (Cole, 2005; Kaye, 2003). One judge argued that a lack of scientific testing and peer review in support of fingerprint evidence should be cause for excluding fingerprint evidence (United States v. Plaza, 2002), although this opinion was later reversed by the same judge. More recently, the National Research Council of the National Academies of Science (2009) called for increased standards in training and documenting the processes by which latent print examiners conduct examinations, as well as for error rate studies to define the limitations of the forensic disciplines.

The most comprehensive study of error rates concluded that while erroneous identifications were relatively rare (about one in every 1000 non-mated pairs was called an identification), erroneous exclusions constituted about 8% of all mated pairs (Ulery, Hicklin, Buscaglia, & Roberts, 2011). This particular study replicated the workflow of casework, in that it allowed examiners to say “no value” for latent prints they deemed of insufficient quality for comparison. As a result, the error rate they measured is not directly comparable to that of forced-choice procedures (see Thompson, Tangen, & McCarthy (2013) for more discussion on this point). In a subsequent study, experts demonstrated fairly high reproducibility, repeating their prior conclusion in about 90% of their decisions (Ulery, Hicklin, Buscaglia, & Roberts, 2012). Although error rate studies bear on the accuracy and reliability of examiners in conditions that are designed to approximate casework, they do not offer mechanistic explanations of the development of expertise or offer a comparison with laypersons to illustrate whether experts in fact differ in their abilities.

Prior work with fingerprint examiners found evidence for information integration across different regions using a behavioral task that briefly presented fingerprint fragments to examiners in a forced-choice design (Busey & Vanderkolk, 2005). The accuracy results were modeled using a probability summation model which demonstrated that accuracy on full images was greater than that expected from performance on the two individual halves. These results suggest examiners may perceptually integrate information over separate regions of an impression when comparing fingerprints. A second experiment demonstrated that the N170 component of the EEG signal was modulated by a 180° rotation of the fingerprint, which is similar to that observed when faces are rotated. However, the evidence for holistic processing in each of these experiments was somewhat indirect, leaving open the question of what information is integrated and whether this integration is automatic or under voluntary control.

In the present work, we directly address the concept of holistic processing using the composite task (Hole, 1994; Young et al., 1987). This task measures our tendency to process objects as a unified whole rather than a compilation of individual parts and has become a common technique to assess the degree to which an irrelevant stimulus half will interfere with the decision based on the relevant half (Richler & Gauthier, 2014). The advantage of this approach is that allows us to infer the nature of information integration across regions without relying on self-report procedures. Examiners may not understand what information they rely on, in part because it may reside below the level of conscious awareness (Ahissar & Hochstein, 1997; Snodgrass, Bernat, & Shevrin, 2004).

**Fig. 1:**
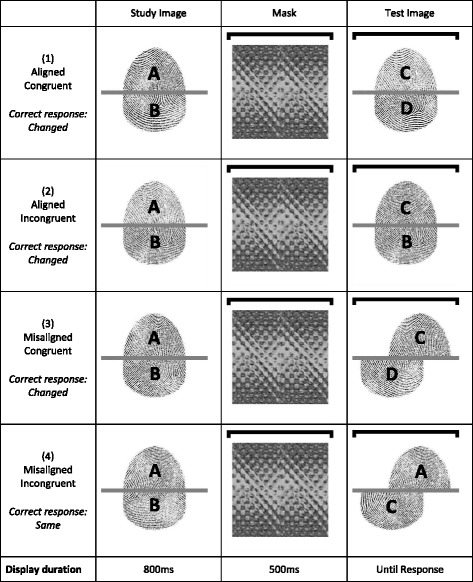
Four example trials with side cued as top. Each *row* is a trial where the stimuli are presented in sequence from left to right. This shows all possible combinations of alignment and congruency. *Capital letters* are used to label the halves for added clarity in this example but were not shown in the actual stimuli

We adapted the composite task for fingerprints as illustrated in Fig. 1. On each trial the observer must attend and encode both the top and bottom halves of a briefly presented fingerprint. This is replaced with a mask and then a second fingerprint is presented. One half is cued during the mask and second image and the observer must respond “same” or “changed” to only the cued half. If the to-be-ignored half is still automatically processed to the point of making a separate “same”/“changed” decision, this decision may facilitate the response to the cued half (if the decisions for each half are congruent) or interfere with the response to the cued half (if the decision for each half are incongruent). Thus, if the participant is processing the image holistically to the point where he or she cannot ignore the uncued half, we will see performance differences between trials where the two halves have decisions that are congruent verses incongruent. Of course, successfully ignoring the uncued half will eliminate the facilitory or inhibitory effects, resulting in no performance difference between congruent and incongruent trials.

As a further test of holistic processing, half of the trials have test images that are misaligned, as shown in rows 3 and 4 of Fig. 1. Here we assume that the physical misalignment breaks the gestalt cue of continuity and the perceptual grouping of the two halves, potentially disrupting holistic processing and therefore reducing the interference from the uncued to-be-ignored half (Richler, Mack, Gauthier, & Palmeri, 2009). Other forms of misalignment, such as the background color of the stimulus, have also been shown to disrupt holistic processing (Curby, Goldstein, & Blacker, 2013). This would produce similar performance between congruent and incongruent trials, because the congruent trials no longer benefit from facilitation from the uncued half.

The combination of large congruency effects for aligned trials and small congruency effects for misaligned trials results in a central signature of holistic processing in the composite task: a congruency × alignment interaction. This interaction is typically measured with d’ (Macmillan & Creelman, 2004) by combining the accuracy from same and changed trials using traditional signal detection theory approaches. The exact nature of this interaction will depend on whether misalignment also produces a performance decrement or whether partial integration still occurs during the misaligned condition. The expected signature of holistic processing is greater performance differences between congruent and incongruent trials for the aligned trials when compared with the difference between congruent and incongruent trials for the misaligned trials.

Why might we expect holistic processing with fingerprints in our latent print examiners? While holistic processing as measured by the composite task was originally characterized as a face-specific mechanism, there is now ample evidence that holistic processing reflects certain classes of perceptual expertise more generally. For example, holistic processing has been observed with expertise for a range of objects such as musical notation (Wong & Gauthier, 2010) and cars (Curby et al., 2013) and develops in the laboratory with expertise for novel objects (Gauthier & Tarr, 2002). The exact mechanisms underlying the emergence of holistic processing in various domains of expertise is the subject of continued debate, with the nature of the interference argued to lie somewhere between a purely perceptual (e.g. template match) account and a purely cognitive (e.g. decision conflict) account, and may rely on the allocation of attention across the two halves (Richler et al., 2012).

Why might we not expect holistic processing? One common feature of domains of expertise that have shown holistic processing is that they require people to individuate objects, which often involves learning names of individuals (Chua, Richler, & Gauthier, 2015; Wong, Palmeri, & Gauthier, 2009). Naming is a natural part of many forms of expertise (e.g. car or bird experts or familiar face recognition). However, fingerprints are rarely named, other than broad classifications based on their overall pattern type (e.g. whorl or arch). The present experiments use an over-learned stimulus set (at least for experts) for which naming is not an integral component of their expert behavior. We avoid any broad naming based on pattern type because when we changed one or both halves of the image, we always replaced the half with the same pattern type (left or right loop); naming at the pattern type level could not contribute to performance in our task.

To test holistic processing with fingerprint examiners, we used the composite task with artificial fingerprint images that are similar to fairly high quality casework images. We chose these images because we did not want subjects to use irrelevant dimensions such as deposition pressure or amount of ink to make same/changed decisions. We ran the composite task on experts and novices using upright fingerprints (Experiment 1) and then tested a new set of experts and novices with inverted fingerprints (Experiment 2). Our results demonstrate the conditions under which congruency effects are observed (or not) by using stimuli that are overlearned but not typically named by examiners. We also address the practical question of whether holistic processing plays a significant role in fingerprint expertise and our comparisons with novices document the degree to which any holistic processing effects are specific to experts. This latter question has implications for the debate about whether examiners have true expertise to contribute beyond showing the images to the jury, as well as how they might attain their superior performance.

## Methods

### Participants

We used two between-subject conditions (expertise and orientation) and as a result there were four different groups of subjects. For Experiment 1, we recruited 14 fingerprint examiners (age range, 31–56 years; mean age, 38.3 years) from one regional, five state, and eight metro forensic science laboratories. Their years of experience were in the range of 5–15 years, with a mean of 8.4 years and an interquartile range of 6–10 years. The novice group for Experiment 1 was made up of 16 people from the Bloomington, Indiana community, but data from one were excluded due to poor accuracy, resulting in 15 people (age range, 18–63 years; mean age, 34.9 years). For Experiment 2, an additional group of 13 fingerprint examiners (age range, 31–62 years; mean age, 40.3 years) were recruited from six state and six metro agencies and one national forensic science lab, with their average years of experience in the range of 2–31 years, with a mean of 9.3 years and an interquartile range of 4–12 years. Another group of 15 novices (age range, 20–58 years; mean age, 35.3 years) were recruited from the Bloomington, Indiana community. All of the novices across both experiments had no prior experience with fingerprints, while experts were required to have at least two years of unsupervised casework experience. All participants were asked to not participate if they were unable to read the text at their comfortable viewing distance. The novices received US$10 in compensation.

### Stimuli

The stimuli for both experiments were composed in the same way, but in Experiment 2, all stimuli were rotated 180°.

Figure 1 illustrates the stimuli used on various trials. The program SFinGe, a synthetic fingerprint generator (Cappelli, Lumini, Maio, & Maltoni, 2007), was used to create a database of 20 left loop fingerprints and 20 right loop fingerprints. We used synthetic fingerprints because real fingerprints have variations in the texture and ink deposition that are idiosyncratic and not particularly relevant during fingerprint comparisons. The SFinGe program has been used to create large databases of stimuli to test automated fingerprint identification systems and it has generally been accepted in certain roles as a proxy for real fingerprints.

These images were cut into two halves and the halves were recomposed in various different combinations to create composites, which were randomized for every trial. The randomization was done so that it was not possible for the top and bottom of the same print to appear together on any study or test image. To accomplish this, four separate images were chosen for each trial. If both top and bottom images did not change, only two images were actually used. If only the top or bottom changed, a third image was used to create the changed half. If both the top and bottom changed, parts of all four images were used to create the study and test images. These four images were always drawn from either the pool of left loops or right loops in order to preserve the overall appearance of a reasonable fingerprint.

A horizontal gray bar, 20 pixels wide, was centered between the two halves to mask the disparities between their ridges. The MATLAB program then added a black bracket cue to either the top or bottom of all the test images and randomly determined if the test image was to be misaligned or aligned. This cue also appeared on the mask. For the misaligned images, the top half was shifted to the right by approximately 35% of its width and the bottom half was shifted to the left by 35% of its width. In the case of Experiment 3, the whole image was rotated 180°, but this was done before the addition of the black cue bracket. The final images were grayscale 562 × 640 pixel .jpeg files. A mask and a fixation point were also created with the same dimensions. These images were saved to the server and loaded by a Javascript program to display the trials, as described below.

### Procedure

The experiment was administered entirely online so we could recruit fingerprint examiners from across the country. Novices also participated using the same online system, either using their own computers or sometimes coming into our laboratory to complete the study on our computer for convenience. Using Javascript, PHP, and HTML, the website was set up so participants could log in using a unique ID and password and complete the study at any pace they desired. All groups of participants performed the same task and all had the option to pause and resume the study at any point.

After a participant logged in, they were presented with a detailed page of instructions, an optional instructional video, and a mandatory set of 16 practice trials to acquaint them with the task. The instructional video did not provide extra information beyond the written instructions; however, participants were encouraged to watch it for further clarification. During a follow-up email, no participants expressed confusion over the procedures of the task. We used the jsPsych library (de Leeuw, 2015) to present the trials within Javascript. For each trial, the participant was shown a study image for 800 ms (2000 ms if it was a practice trial), then a random pattern mask for 500 ms, and finally a test image that was shown until the participant responded. If their response took longer than 2500 ms, the participant was cautioned and asked to respond faster on subsequent trials. Internal timing within Javascript captured the display durations, which was used to screen out computers with improper timing (however, no participants were excluded for this reason). The process and layout of the trials is visualized in Fig. 1.

A black bracket would first appear on either the top or bottom of the mask and then continue to show for the test image, cueing the participant on which half to attend to. Participants were asked to make a response, “same” or “changed,” based on whether the cued half of the test image was the same or different from the study image. The study image would always be aligned and the test image would be either aligned or misaligned. This response was then recorded to a MySQL database via PHP and the next trial would begin. The participant would receive feedback after every 16 trials, telling them the percent of answers they got correct during these recent trials. There were 20 trials for each combination of cued half (top or bottom), trial type (match or non-match), side congruency (congruent or incongruent), and alignment (aligned or misaligned), resulting in 320 trials total. These trials do not include the 16 practice trials that participants were given during instruction; however, within the practice trials each combination of the four conditions was presented once. The order of conditions during the trials was randomized and conditions were not blocked. We allowed the participants to stop at any particular trial and resume the study at their leisure.

### The task

Experiment 1 and Experiment 2 were both 2 × 2 × 2 mixed designs, where the within-subjects factors were side congruency (congruent or incongruent) and alignment (halves were aligned or misaligned). The between-subjects factor was expertise (expert or novice). Experiments 1 and 2 were differentiated only by the orientation of the fingerprints (upright for Experiment 1 or inverted for Experiment 2) and this manipulation was also between-subjects.

Based on the composite task, the halves of the stimuli were congruent if the decision (“same” or “changed”) for the uncued half was the same as the decision for the cued half. The halves were incongruent if they led to different decisions and according to the logic of the composite task, this incongruency should cause the uncued half to interfere with the cued half if the image is being processed holistically. Note that “congruency” here refers to the decisions for the two halves, not whether either was changed. For example, if both the cued and uncued halves changed, this would still be considered a congruent trial because the decision is the same for both the cued and uncued halves. The misaligned images should in theory hinder any holistic processing and reduce the interference of the incongruent side, due to the increased physical disjunction of the two halves.

## Results and discussion

Results for the composite task are typically presented in terms of accuracy (d’) and reaction time, with accuracy receiving more attention in the literature. We computed d’ in the same way for each combination of alignment and congruency, by calculating the normal-transformed hit rate (the proportion of times each participant said “same” when the ground truth was “same”) and subtracting from it the normal-transformed false alarm rate (the proportion of times each participant said “same” when the ground truth was “changed”). Although participants were cautioned to respond faster if their response took longer than 2500 ms, we did not exclude these trials from analysis. Such trials represented only 2.3% of the overall dataset, but five subjects out of our entire set of 57 subjects had greater than 10% of their trials fall into this category. If these trials are excluded, we find similar results to those reported below with the exception of the experts-only congruency by alignment interaction in Experiment 1.

### Experiment 1: upright fingerprints

#### Sensitivity (d’)

**Fig. 2:**
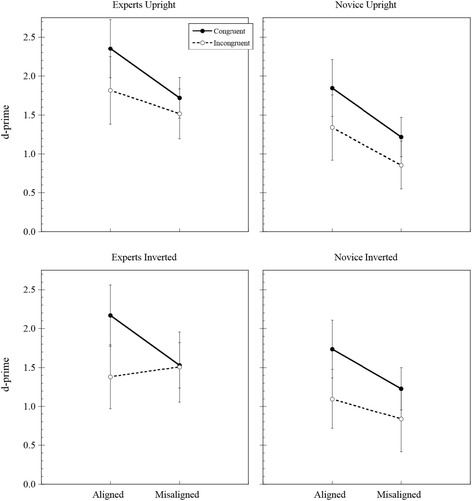
d-prime (sensitivity) values for both experiments. The two *top panels* represent Experiments 1 (upright prints) and the two *bottom panels* represent Experiment 2 (inverted prints). *Error bars* are 95% confidence intervals computed from the standard error of the mean

The two top panels of Fig. 2 show the d’ value for each of the four conditions (two levels of alignment crossed with two levels of congruency) separated into two graphs by expertise. The standard marker of holistic processing is a relatively large difference between the congruent and incongruent d’ values when the halves are aligned, and a reduced difference when the halves are misaligned (Richler & Gauthier, 2014). In other words, the incongruent, task-irrelevant halves should interfere with performance more when the halves are aligned than when they are misaligned.

A repeated-measures, mixed-factor 2 (congruency: congruent, incongruent) × 2 (alignment: aligned, misaligned) × 2 (expertise: expert, novice) analysis of variance (ANOVA) was performed for experiment 1. The ANOVA revealed a strong main effect of expertise with *F*_1,27_ = 7.98, *MSE* = 1.042, *p* < 0.01, and *η*_*p*_^*2*^ = 0.228, confirming that experts are more sensitive to the task overall. There was a main effect of congruency (*F*_1,27_ = 15.21, *MSE* = 0.308, *p* < 0.01, *η*_*p*_^*2*^ = 0.360), and this effect was not different between experts and novices (*F*_1,27_ = 0.091, *MSE* = 0.308, *p* = 0.765, *η*_*p*_^*2*^ < 0.01) and a main effect of alignment (*F*_1,27_ = 38.93, *MSE* = 0.195, *p* < 0.01, *η*_*p*_^*2*^ = 0.590), which was also not different across experts and novices (*F*_1,27_ = 0.295, *MSE* = 0.195, *p = 0.591*, *η*_*p*_^*2*^ = 0.011). These main effects of congruency and alignment, where d’ is generally lower in all incongruent trials and in all trials where the test image is misaligned, are consistent with other work involving the composite task (Chua et al., 2015; Richler, Bukach, & Gauthier, 2009; Richler, Mack, Palmeri, & Gauthier, 2011). There was a trend-level interaction between congruency and alignment overall (*F*_1,27_ = 3.76, *MSE* = 0.110, *p* = 0.063, *η*_*p*_^*2*^ = 0.122), but this slight interaction was not significantly different across expertise (*F*_1,27_ = 0.572, *MSE* = 0.110, *p* = 0.456, *η*_*p*_^*2*^ = 0.021). A significant interaction between congruency and alignment signifies holistic processing in the composite task and one priori hypothesis was that this interaction would vary across latent fingerprint expertise. Separate two-way ANOVAs were done to unpack the expert and novice groups, which revealed a small but significant interaction between congruency and alignment for experts (*F*_1,13_ = 5.000, *MSE* = 0.077, *p* = 0.0435, *η*_*p*_^*2*^ = 0.278).^1^ We found no significant interaction between congruency and alignment for novices (*F*_1,14_ = 0.564, *MSE* = 0.080, *p* = 0.465, *η*_*p*_^*2*^ = 0.039). However, given a lack of significant three-way interaction between congruency, alignment, and expertise, the significant two-way interaction between congruency and alignment for experts and the lack of thereof for novices should not be viewed as compelling evidence for differences in holistic processing due to expertise in upright fingerprints.

#### Response bias (criteria)

**Fig. 3:**
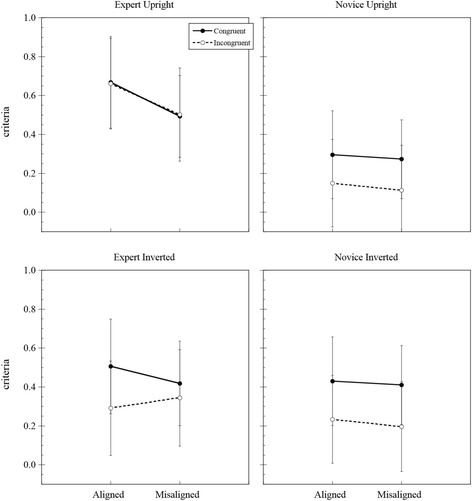
Criteria plotted for both experiments. The two *top panels* represent Experiments 1 (upright prints) and the two *bottom panels* represent Experiment 2 (inverted prints). *Error bars* are 95% confidence intervals computed from the standard error of the mean

The two top panels of Fig. 3 show the response bias (criterion) values for Experiment 1 in both experts and novices. Criterion was calculated by normal-transforming the result of 1 minus the false alarm rate for each condition. The predictions for the holistic model are less clear for the criteria values, in part because they depend on complex factors such as probability matching (the tendency for participants to want to give an equal rate of “same” and “different” responses over the course of the experiment), which can interact with shifting underlying distributions as demonstrated by differences in d’. However, they do document whether participants tend to adopt more liberal or conservative response strategies for different levels of congruency or alignment. We performed a similar three-way ANOVA on criteria and only found a main effect of expertise with *F*_1,27_ = 8.44, *MSE* = 0.478, *p* < 0.01, and *η*_*p*_^*2*^ = 0.238. An additional ANOVA on the log beta values revealed no main effect for expertise however, showing that experts were more conservative primarily because they also had higher d’ values and adjusted their criteria accordingly.

#### Response times

**Fig. 4:**
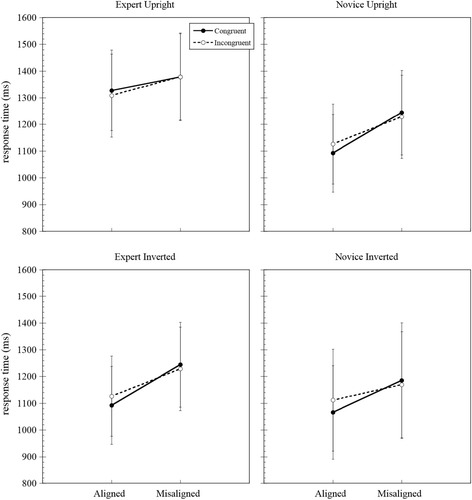
Mean response times plotted for both experiments. The two *top panels* represent Experiments 1 (upright prints) and the two *bottom panels* represent Experiment 2 (inverted prints). *Error bars* are 95% confidence intervals computed from the standard error of the mean

The two top panels of Fig. 4 show the mean reaction times for all trials in each of the eight conditions for Experiment 1. Holistic processing is sometimes observed in response times, but not always (Curby et al., 2013; Wong et al., 2009). Examining response times also demonstrates when participants may have made speed/accuracy tradeoffs. The reaction times showed a main effect of alignment (*F*_1,27_ = 17.102, *MSE* = 14911, *p* < 0.01, *η*_*p*_^*2*^ = 0.388) and a trend-level effect of expertise (*F*_1,27_ = 3.003, *MSE* = 295695, *p* = 0.094, *η*_*p*_^*2*^ = 0.100). There were no significant interactions between any variables (all *p* values > 0.15). The lack of interactions suggests that participants did not operate in vastly different speed/accuracy tradeoff regimes.

#### Summary

Overall, there is weak evidence for holistic processing as an element of expertise matching upright fingerprints. Although the interaction between congruency and alignment for d’ may appear stronger for experts, there was no significant difference when compared with the novice interaction. As expected, experts are better at the task in general, but this is difficult to attribute to holistic processing. These results contrast with other domains such as musical notation (Wong & Gauthier, 2010), cars (Curby et al., 2013), faces (Richler & Gauthier, 2014), and novel objects (Gauthier & Tarr, 2002), which demonstrated clear evidence for holistic processing.

### Experiment 2: inverted fingerprints

As a further test of the hypothesis that fingerprint examiners would demonstrate holistic processing for fingerprints, we repeated Experiment 1 with a new group of experts and novices, but instead used inverted stimuli. Stimulus inversion is commonly used to disrupt holistic processing, as seen in the familiar Thatcher Illusion (Thompson, 1980). However, even with faces, the effects of inversion remain somewhat complicated, with some authors arguing that inversion does not qualitatively change the nature of perceptual processing (e.g. Sekuler, Gaspar, Gold, and Bennett, 2004). Richler et al. (2011) manipulated exposure duration and inversion of faces to identify the time-course of holistic processing effects and found that holistic processing was present for inverted faces but that it emerged later compared to upright faces. With fingerprints, Thompson and Tangen (2014) manipulated orientation and found no performance difference during a 60-s side-by-side comparison of fingerprints corrupted by noise. This result seems in contrast to from the conclusion drawn by Busey and Vanderkolk (2005), who argued that fingerprint inversion produced changes in the N170 component that were consistent with those seen when faces are inverted.

Experiment 2 is designed to assess whether inversion affects the holistic processing of fingerprints by experts specifically with the composite task, so it replicated and extended Experiment 1 with new observers and inverted fingerprints. Otherwise all procedures were identical.

#### Sensitivity (d’)

The two bottom panels of Fig. 2 show the d’ value for each of the four conditions separated by expertise. A clear interaction is present in the expert data, where misalignment completely eliminates any interference caused by the incongruent un-attended half. The repeated-measures, mixed-factor 2 (congruency: congruent, incongruent) × 2 (alignment: aligned, misaligned) × 2 (expertise: expert, novice) ANOVA confirmed this result.

With inverted fingerprints, there was only a trend-level effect of expertise with *F*_1,26_ = 3.424, *MSE* = 1.442, *p* = 0.076, and *η*_*p*_^*2*^ = 0.116. There was a main effect of congruency (*F*_1,26_ = 29.586, *MSE* = 0.200, *p* < 0.01, *η*_*p*_^*2*^ = 0.532) and this effect was not different between experts and novices (*F*_1,26_ = 0.422, *MSE* = 0.200, *p* = 0.522, *η*_*p*_^*2*^ = 0.016) and a main effect of alignment (*F*_1,26_ = 22.673, *MSE* = 0.126, *p* < 0.01, *η*_*p*_^*2*^ = 0.466), which was also not different across expertise (*F*_1,26_ = 0.903, *MSE* = 0.126, *p = 0.351*, *η*_*p*_^*2*^ = 0.034). These main effects of congruency and alignment are very similar to Experiment 1.

There was a significant interaction between congruency and alignment (*F*_1,26_ = 16.204, *MSE* = 0.110, *p* < 0.01, *η*_*p*_^*2*^ = 0.384) and a trend-level interaction between congruency, alignment, and expertise (*F*_1,26_ = 4.207, *MSE* = 0.110, *p* = 0.050, *η*_*p*_^*2*^ = 0.139) which supports our initial observation of the graphs. Recall that this three-way interaction between congruency, alignment, and expertise that is predicted by the holistic processing hypothesis (and was not significant in Experiment 1). To specify the exact interaction that varies across expertise, we again divided the three-way ANOVA into separate two-way ANOVAs by expertise. For the expert group, there was a significant interaction between congruency and alignment (*F*_1,12_ = 23.924, *MSE* = 1.896, *p* < 0.01, *η*_*p*_^*2*^ = 0.666); for the novice group, there was not a significant interaction between congruency and alignment (*F*_1,14_ = 1.692, *MSE* = 0.231, *p* = 0.214, *η*_*p*_^*2*^ = 0.108). With inverted fingerprints, we see that experts find it more difficult to ignore the task-irrelevant half when it is incongruent and conflicts with the response they should be making, leading to a decrease in d’ for those trials. When the halves are misaligned, however, this interference is eliminated as shown in the lower-left panel of Fig. 2. This interaction was not present in novices and our three-way ANOVA confirms this with the trend-level interaction (*p* = 0.050). Possible reasons of this stronger interaction for inverted prints compared to upright prints are discussed in the “General discussion”.

#### Response bias (criteria)

The two bottom panels of Fig. 3 show the response bias (criterion) values for Experiment 2 in the eight conditions. We performed a similar three-way ANOVA on criteria and found a main effect of congruency with *F*_1,26_ = 9.836, *MSE* = 0.086, *p* < 0.05, and *η*_*p*_^*2*^ = 0.274. No additional main effects or interactions between any variables were found (*p* values all > 0.32).

#### Response times

The two bottom panels of Fig. 4 show the mean reaction times for all trials for each of the eight conditions for Experiment 2. The three-way ANOVA showed the main effect of alignment (*F*_1,26_ = 31.857, *MSE* = 7778, *p* < 0.01, *η*_*p*_^*2*^ = 0.551) and an interaction between congruency and alignment (*F*_1,26_ = 7.573, *MSE* = 23987, *p* = 0.011, *η*_*p*_^*2*^ = 0.226), but this interaction did not interact with expertise (*F*_1,26_ = 0.018, *MSE* = 3167.5, *p* = 0.895, *η*_*p*_^*2*^ < 0.01). This interaction shows that both novices and experts were faster when the halves were both congruent and aligned. There were no other main effects or significant interactions between any variables (all *p* values > 0.27).

### Cross-experiment analysis

We examined d-prime values once more with a repeated measure, mixed-factor 2 (congruency: congruent, incongruent) × 2 (alignment: aligned, misaligned) × 2 (expertise: expert, novice) × 2 (orientation: upright, inverted) ANOVA to look for effects and interactions across Experiments 1 and 2. There was a between-subjects main effect of expertise (*F*_1,53_ = 10.496, *MSE* = 1.238, *p* < 0.01, *η*_*p*_^*2*^ = 0.165), but no main effect of orientation (*F*_1,53_ = 1.008, *MSE* = 1.238, *p* = 0.320, *η*_*p*_^*2*^ = 0.019). This is consistent with Thompson and Tangen (2014), who also found no effect of inversion on the accuracies of experts or novices. There was also a main effect of congruency (*F*_1,53_ = 41.462, *MSE* = 0.255, *p* < 0.01, *η*_*p*_^*2*^ = 0.439) and a main effect of alignment (*F*_1,53_ = 61.022, *MSE* = 0.161, *p* < 0.01, *η*_*p*_^*2*^ = 0.535) as well as an interaction between congruency and alignment (*F*_1,53_ = 17.891, *MSE* = 0.110, *p* < 0.01, *η*_*p*_^*2*^ = 0.252). While this is consistent with the holistic processing hypothesis, combining across both upright and inverted fingerprints, the congruency by alignment by expertise three-way interaction was only of trend-level significance (*F*_1,53_ = 3.974, *MSE* = 1.110, *p* = 0.051, *η*_*p*_^*2*^ = 0.070), which can be interpreted as weak support for holistic processing as a function of expertise. Aside from a trend-level interaction between alignment and orientation (*F*_1,53_ = 3.224, *MSE* = 0.161, *p* = 0.078, *η*_*p*_^*2*^ = 0.057), there were no other main effects or interactions between factors (*p* > 0.13).

## Conclusions

In Experiment 1, we measured holistic processing in experts and novices for upright fingerprints and found no significant difference between the groups. In Experiment 2, we measured holistic processing for inverted fingerprints to test if inversion could affect holistic processing and found that the interaction between congruency, alignment, and expertise was stronger than what was observed with upright fingerprints. The cross-experiment analysis revealed trend-level interactions that provide weak support for holistic processing as a function of expertise, because the congruency by alignment interaction of experts is only marginally greater than the same interaction in novices.

Despite this relatively weak support for holistic processing in experts being significantly greater than that in novices, there is still support for holistic processing in the experts alone. The unpacked ANOVAs show that the signature congruency by alignment interaction is present in experts for both upright and inverted prints, which is consistent with other domains of expertise like musical notes, faces, or cars. Novices may also show some evidence for holistic processing as evidenced by the slight non-parallelism in the right panels of Fig. 2, which may be sufficient to reduce the congruency by alignment by expertise interaction to trend level. Novices may have some level of natural expertise when it comes to fingerprints. Additionally, SFinGe generates the fingerprints with a standard oval shape, giving them an overall form-factor that is similar to what participants may be used to processing in faces. These factors may contribute to the relatively small differences between experts and novices in holistic processing. SFinGe also generates impressions that lack strong cues to orientation that are produced by pressure differences and the lack of these cues may have resulted in experts relying on different features than they would for natural prints.

Given that our results show only modest levels of holistic processing across expertise and that inversion did not have the usual effect, one possibility is that fingerprint experts may not utilize the same perceptual mechanisms as experts in other domains. The most apparent difference between fingerprint examination and other domains is that fingerprint experts do not typically name or individuate specific fingerprints outside their basic pattern class (such as loop or whorl). Cars can be individuated by the model, type, and style, faces can be recognized based on the name of the person and other attributes such as gender or race, and even made-up novel objects such as greebles or ziggerins are named as a test for perceptual expertise. In addition, holistic effects may only occur if the training is face-like, as opposed to letter-like (Gauthier & Tarr, 1997; Wong et al., 2009). Fingerprints are different in that they are an over-learned area of expertise that does not require naming and, as a result, holistic processing as it is defined by the composite task may be less pronounced. An alternative hypothesis we cannot exclude is that our sample size was not large enough to produce robust evidence for holistic processing differences due to expertise.

Why might we see stronger evidence for holistic processing for inverted stimuli relative to upright stimuli? Given that the literature suggests we find the opposite, we have no clear account. However, besides the usual possibility of sampling error, we do note that experts encounter fingerprints in various orientations during casework. They usually immediately orient them upright and then spend a great deal of time looking at the upright version. However, most of the exposure to upright prints involves the engagement of more localized feature processing such as minutiae comparisons, which could be a more analytic process based on local features. However, inverted fingerprints usually involve the extra step of first orienting the print. The appearance of an inverted fingerprint might engage a more holistic process that is involved in deciding whether and how to orient the print to an upright state. At this point, such an account must remain somewhat speculative as the literature does not offer a precedent for stronger holistic effects for inverted stimuli.

Taken together, the present results generalize the range of tasks where holistic processing has been measured and indicate differences between fingerprint examination and other domains of expertise.

The results also have implications for practitioners. We see overall better performance on the task for experts than novices, which is consistent with perceptual expertise for these examiners. Even though these holistic effects appear relatively weak, they may still contribute to an examiner’s expertise in extended casework comparisons by facilitating rapid integration across image regions and this is consistent with the idea that fingerprint examiners have expertise to share during a trial that goes beyond the perceptual processing skills of a novice sitting on a jury. However, examiners should be careful not to assume that these perceptual skills make them invulnerable to errors or replace the hard work of detailed and lengthy perceptual examinations of some latent prints.

## Footnotes

1 This interaction was the only result that was affected when excluding trials with response times greater than 2500 ms. It became trend-level significant (F1,13 = 3.504, MSE = 0.093, p = 0.0839, *η*_*p*_^*2*^ = 0.212).
